# Quantifying the density and utilization of active sites in non-precious metal oxygen electroreduction catalysts

**DOI:** 10.1038/ncomms9618

**Published:** 2015-10-21

**Authors:** Nastaran Ranjbar Sahraie, Ulrike I. Kramm, Julian Steinberg, Yuanjian Zhang, Arne Thomas, Tobias Reier, Jens-Peter Paraknowitsch, Peter Strasser

**Affiliations:** 1 Department of Chemistry, The Electrochemical Energy, Catalysis and Material Science Laboratory, Chemical Engineering Division, Technical University Berlin, Straße des 17. Juni 124, 10623 Berlin, Germany; 2 Department of Chemistry, Technical University Darmstadt, Jovanka-Bontschits-Street 2, 64287 Darmstadt, Germany; 3 Department of Materials and Earth Science, Technical University Darmstadt, Jovanka-Bontschits-Street 2, 64287 Darmstadt, Germany; 4 School of Chemistry and Chemical Engineering, Southeast University, Nanjing 211189, China; 5 Department of Chemistry, Division of Functional Materials, Technical University Berlin, Hardenbergstr. 40, 10623 Berlin, Germany

## Abstract

Carbon materials doped with transition metal and nitrogen are highly active, non-precious metal catalysts for the electrochemical conversion of molecular oxygen in fuel cells, metal air batteries, and electrolytic processes. However, accurate measurement of their intrinsic turn-over frequency and active-site density based on metal centres in bulk and surface has remained difficult to date, which has hampered a more rational catalyst design. Here we report a successful quantification of bulk and surface-based active-site density and associated turn-over frequency values of mono- and bimetallic Fe/N-doped carbons using a combination of chemisorption, desorption and ^57^Fe Mössbauer spectroscopy techniques. Our general approach yields an experimental descriptor for the intrinsic activity and the active-site utilization, aiding in the catalyst development process and enabling a previously unachieved level of understanding of reactivity trends owing to a deconvolution of site density and intrinsic activity.

## Abstract (web summary)

Iron and nitrogen doped carbon materials are widely studied electrocatalysts, however measurement of features such as intrinsic turn-over frequency and active site utilization has proved difficult. Here, the authors use a combination of chemisorption and spectroscopy techniques to determine these properties.

## Graphical abstract

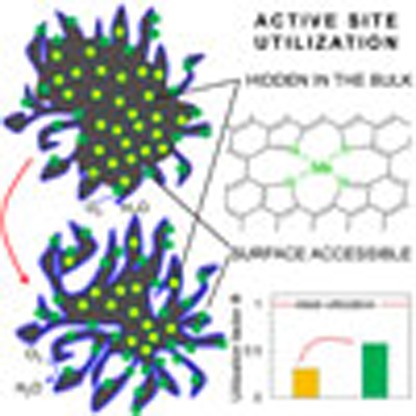

The electrochemical reduction of oxygen (‘oxygen reduction reaction', ORR) in acid and alkaline environments is a tremendously important reaction for a variety of emerging electrochemical energy technologies and device components such as cathodes of metal air batteries^1^ and fuel cells^2^,^3^,^4^,^5^, or oxygen depolarization cathodes in brine and hydrochloric acid electrolysers^6^. However, sluggish ORR kinetics causes prohibitively high-energy efficiency losses. Presently, platinum and its alloys are widely used in fuel cells; however, the scarcity of Pt calls for a replacement of this costly noble metal with less expensive metals (Me) such as iron, cobalt and manganese in the form of Me–N–C catalysts^7^,^8^,^9^. To achieve this goal, a rational design of non-precious metal catalysts (NPMC) with superior activity over Pt materials and stability has attracted much attention^2^,^5^,^10^,^11^,^12^. Recently, significant advances have been made in improving the activity of NPMCs to the levels comparable to Pt-based materials^2^,^13^. Evidence is mounting that the key structural motif of these catalysts are nitrogen-coordinated transition metal ions of MeN_*x*_ (*x*=2…4) embedded in basal planes of carbon or bridging two graphene planes at their edges^8^,^14^,^15^,^16^,^17^,^18^,^19^,^20^. NPMCs still suffer from poor durability^2^,^4^,^11^,^21^,^22^, which is in part attributed to corrosion of the carbon support and the active sites by by-products such as H_2_O_2_. Accordingly, current key goals in non-precious metal ORR catalysis comprise molecular insight in the catalytically active site^15^,^23^, optimized synthesis and interrogation tools for the active-site density, as well as strategies to stabilize the active catalytic sites during oxygen reduction^5^,^9^,^24^,^25^. In this respect, particular attention was placed on a specific family of Me-N-C catalysts derived from high-nitrogen content molecules, such as cyanamide and from heterocyclic polymers such as polyaniline (PANI), or combinations thereof^5^,^9^,^25^.

A recent report by Zelenay and co-workers^5^ addressed possible benefits of a bimetallic active site to improve the final stability of the catalysts. They showed that simultaneous utilization of Co and Fe precursors can increase the stability of the catalyst in acid electrolyte.

Our incomplete understanding of the role of the metal ion in the active-site structure originates in large parts from the lack of a suitable technique to probe the chemical composition, density and reactivity of the active sites in these types of catalysts. Molecular probes, such as cyanide and carbon monoxide, have been used in a number of unsuccessful efforts to poison active catalytic sites in rotating disk electrode (RDE) measurements^26^,^27^,^28^. At room temperature results showed no measurable deactivation effect on catalyst performance in the presence of CO as typically seen for Pt-containing catalysts. Hence, probing active sites under RDE condition using CO has not been a viable strategy to probe this type of NPMCs.

In the present work, we use PANI-derived Me–N–C catalyst materials as model systems to demonstrate the benefit of a combined active-site analysis by Mössbauer spectroscopy and CO pulse chemisorption/desorption. Unlike earlier studies, low-temperature CO pulse chemisorption and temperature-programmed desorption (TPD) are successfully applied as chemical interrogation tools to obtain quantitative insights into the density of active adsorption sites of the catalysts. To the best of our knowledge, this is the first time that pulse chemisorption and TPD are successfully applied to quantify surface adsorption sites of Me–N–C materials. Our analysis correlates the active-site density with the apparent catalytic reactivity in acid and alkaline media, yielding surface site-based metal-specific catalytic turn-over frequencies (TOFs). In combination with ^57^Fe Mössbauer spectroscopy, we derive a measure for the active-site utilization of the (Fe-based) NPMCs, quantifying the limits of further activity enhancement. Moreover, the direct comparison of TOFs of mono- and bimetallic catalysts uncovers previously unexplored synergistic effects between two dissimilar metal ions in the enhancement of the ORR activity in alkaline.

## Results

### Catalytic oxygen reduction activity and stability

**Figure 1:**
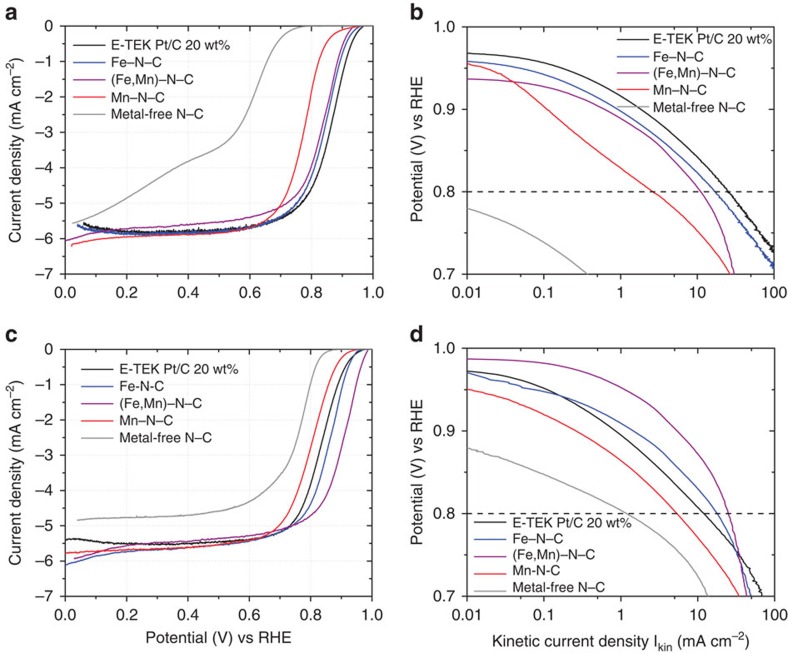
Electrochemical activity evaluation. Linear Scan Voltammetry (LSV) of Me-N–C–3HT–2AL catalysts in oxygen-saturated acid (**a**) and alkaline (**c**) electrolytes. Electrochemical Tafel curves (applied E versus log I_kin_) derived from **a** and **c** in acid, **b** and **d** in alkaline are also given. For reasons of comparison the Me–N–C catalysts are compared with a commercial Pt/C catalyst and the metal-free N–C catalyst. Conditions: RDE experiment at 1,500 r.p.m., in O_2_-saturated 0.1 M HClO_4_ (**a**,**b**) and 0.1 M KOH (**c**,**d**), 25 °C. Geometric catalyst loadings of NPMCs: 0.8 mg cm^−2^, and that of Pt/C: 50 μg cm^−2^ (10 μg_Pt_ cm^−2^). Measurements were repeated three times leading to the same results (Errors are given in Supplementary Tables 1 and 2).

Figure 1 displays the catalytic ORR performances of the Fe–N–C, the Mn–N–C and the bimetallic (Fe,Mn)–N–C catalysts (each after 3 heat treatment (HT) and 2 acid leaching (AL) steps, following our notation) in acid and alkaline electrolytes. The preparation of the catalysts, partially encoded in their notation, is illustrated in Supplementary Fig. 1. The shape and diffusion-limiting currents of the voltammetric sweeps (Fig. 1a,c) are consistent with a four-electron reduction of O_2_ to H_2_O, while their Tafel Plots (Fig. 1b,d) evidence some of the highest catalytic activities ever recorded at low and high pH^5^,^9^,^25^.

The mass activity, onset-potential and half-wave potential of these three catalysts, Me–N–C–3HT–2AL, are listed in Supplementary Tables 1 and 2 together with their mother samples, that is, 2HT–1AL. The ORR activity increased or remained constant with these final AL and HT steps, even though the overall metal content decreased by about 50% for all three catalysts during this treatment (Supplementary Table 3). On a first view, this is consistent with the notion that ORR inactive species are gradually removed by iterative HT–AL cycles. It is further supported by X-ray powder diffraction data in Supplementary Fig. 2: while crystalline sulphides dominate the X-ray pattern after the first HT, all sulphide-related diffraction fingerprints vanished after the final third HT. In the diffractograms, only broad reflexes assigned to a micrographitization of the final catalysts remained visible (Supplementary Fig. 2). In line with this observation, we note that non-precious ORR catalysts reported earlier, derived from singly-leached PANI–Fe–C composites (prepared by a 2HT–1AL cycle), still did contain substantial amounts of residual ORR catalytically inactive crystalline sulphides and thus performed inferior to the present materials^5^,^14^.

**Figure 2:**
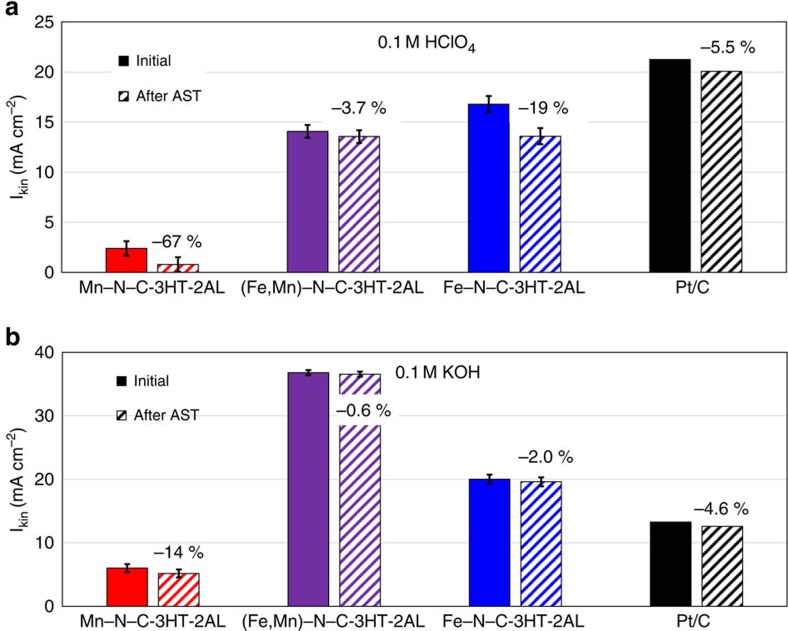
Electrochemical stability evaluation. Kinetic current densities I_Kin_ at 0.8 V (mA cm^−2^) for the oxygen reduction reaction (ORR) of mono- and bimetallic Me–N–C catalysts evaluated (**a**) in acid, 0.1 M HClO_4_ and (**b**) in alkaline, 0.1 M KOH before (solid) and after accelerated stress tests (AST; hatched); Accelerated stress tests involved 9,000 potential cycles between 0.5 and 1.3 V versus RHE with 50 mV s^−1^ in N_2_-saturated electrolyte. Error bars give the s.d. from the overall number of performed experiment.

To assess the stability of the catalysts, we performed a severe accelerated stress test (AST) by cycling the potential between 0.5 V_RHE_ and 1.3 V_RHE_ (see Methods section for further details). The results are summarized in Fig. 2a,b. In acid environment, as expected due to the oxidizing potential of 1.3 V, a substantial decrease in catalytic ORR activity occurred for both monometallic catalysts (−19% for Fe–N–C–3HT–2AL and −67% for Mn–N–C–3HT–2AL). In alkaline environment, both monometallic catalysts performed more stable as in acid environment, with a drop of −2% for Fe–N–C–3HT–2AL and −14% for Mn–N–C–3HT–2AL. In contrast, the bimetallic (Fe,Mn)–N–C–3HT–2AL catalyst exhibited favourable performance stability in both acid (−4%) and alkaline (−1%) environments. We attribute this exceptional stability to a possible synergistic effect brought about by co-doping with two dissimilar transition metals. The electrochemical data in Figs 1 and 2 bear testimony to the promising ORR catalytic performance characteristic of the novel family of bimetallic (Fe,Mn)–N–C materials.

The fuel cell polarization curves, cycling stability plots (O_2_–H_2_) and the relative change in activity in dependence on the cycle number are shown in Supplementary Fig. 3 for our Fe- and (Fe,Mn)–N–C–3HT–2AL catalysts. The trends in activity are in good agreement with RDE measurements (Fig. 1). Comparing our catalyst with today's most active Fe–N–C catalyst published by Jean-Pol Dodelet's group, we have to make the following conclusions: while reaching even higher geometric current densities in RDE conditions^29^, the surface-specific current densities of our catalysts are far of that catalyst in Proton Exchange Membrane Fuel Cell (PEM FC)^2^. This most active Fe–N–C is still not reaching the performance of Pt/C^2^. With having these outstanding performance in RDE, we assume that an optimization of the ink receipt (specifically the Nafion to Catalyst ratio) might enable even more favourable ORR activities in FC too^29^. The change of current as a function of cycle numbers is provided in Supplementary Fig. 3b,c and compared with data taken from Gang Wu *et al*.^5^, for a PANI–Fe–C catalyst degraded with an AST protocol close to ours. As compared with RDE the bimetallic catalyst (Fe,Mn)–N–C–3HT–2AL performs as stable or even more stable compared with Fe–N–C–3HT–2AL (at U>0.8 V). It can be seen that both of our catalysts reveal under cycling conditions a similar stability compared with Wu *et al*. up to 10,000 cycles^5^, while the absolute current density values are smaller.

### Probing catalytically active sites in bulk and surface

**Figure 3:**
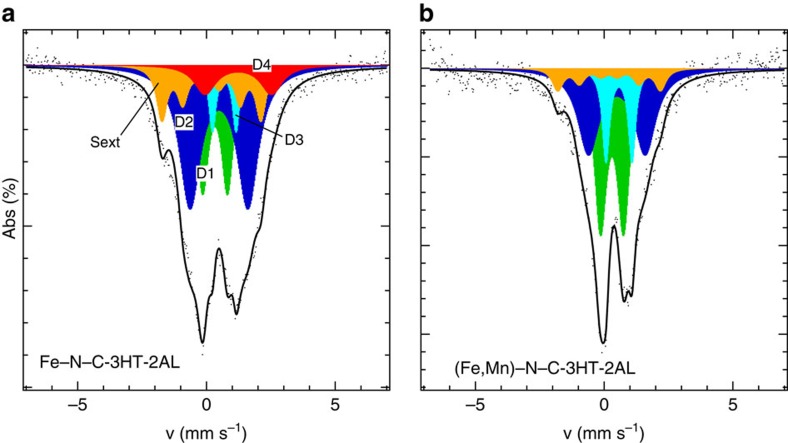
Structural characterization of iron species. ^57^FeMössbauer spectra of (**a**) Fe–N–C–3HT–2AL and (**b**) (Fe,Mn)–N–C–3HT–2AL. The assignment to individual iron sites D_i_ is indicated in **a**. Detailed Mössbauer parameters and Fe concentration of each iron site are summarized in Supplementary Table 4.

To obtain atomic-scale insight into catalytically active Fe centres, the two Fe-containing catalysts were investigated using ^57^Fe Mössbauer spectroscopy^17^,^21^,^22^. Figure 3 shows the Mössbauer spectra of the Fe and bimetallic Fe–Mn catalysts after 3HT–2AL, with Supplementary Table 4 summarizing the experimental Mössbauer parameters with detailed assignments of iron species. For reasons of comparison, their mother samples (2HT-1AL) are given in Supplementary Fig. 5.

The first two doublets are typical for Fe–N–C catalysts^16^,^17^,^20^,^21^,^30^. Their Mössbauer parameters are assigned to a ferrous low-spin FeN_4_ site (D1) and a ferrous intermediate-spin FeN_4_ site (D2). In previous reports, the D1-related site was identified as ORR active site in Fe–N–C catalysts^11^,^16^,^17^,^20^,^21^,^30^. Besides these two doublets, we found two additional doublets and a sextet. These were assigned to a (NFe^III^N_4_)–O_2_ site (D3)^31^, a ferrous high-spin XY-FeN_4_ site (D4, X, Y represent two weak axial ligands as for example, oxygen or nitrogen)^20^ and a small fraction of magnetic particles. The small magnetic field is an indication of reduced particle size (<20 nm)^20^,^32^, whereas its isomer shift indicates that the iron is in an oxidized state (most probable Fe^3+^). Induced by the reduced particle size, these particles appear X-ray amorphous. It should be noted that the Mössbauer parameters of our doublet D4 are close to values previously found for a NFeN_2+2_…NH^+^/C site^20^,^22^. A comparison of the Mössbauer parameters is given in Supplementary Table 5, but illustrates that the isomer shift and quadrupole splitting of our D4 doublet are significantly higher (by 0.2 mm s^−1^ for δ_Iso_ and 0.4 mm s^−1^ for ΔE_Q_). In this case, the higher isomer shift is caused by the additional coordination of our FeN_4_ site by two weak ligands in comparison with the fivefold-coordinated site NFeN_2+2_ in accordance with literature. Assuming that our D4 doublet should be assigned to that particular FeN_2+2_…NH^+^/C site, a significantly larger mass-related activity for the Fe–N–C catalyst versus the bimetallic catalyst would be expected, as only the Fe–N–C catalyst contains this site that is reported as catalytically highly active one. In addition, this catalytic site is ‘famous' for its fast deactivation, whereas all of our catalysts are rather stable in PEM-FC and RDE. Therefore, we assume that our D4 site is indeed different from that site.

With the isomer shifts, δ_iso_, and the quadrupole splittings, ΔE_Q_, for the different iron sites being similar in all catalysts, we conclude that there is no evidence for an alloy-like electronic interaction between iron and manganese. On the other hand, the data evidenced that the two catalysts contained substantially smaller fractions of inorganic Fe species (here only oxidized iron particles) compared with PANI-based catalysts reported earlier^5^,^14^. Those catalysts contained significant fractions of Fe_3_S_4_, iron nitride and iron carbide. We attribute the absence of these phases in our catalysts to the smaller amount of ammonium peroxidisulphate (APS) employed in the synthesis (ratio aniline:APS=3:1 compared with 1:1 for the PANI-based catalysts of Zelenay's group). Consequently, other iron sulphide modifications were formed that were removed more effectively during AL. This effect was previously also found for other Me–N–C catalysts^33^,^34^. The Mössbauer spectra of the two Me–N–C–2HT–1AL catalysts in Supplementary Fig. 5 confirm this interpretation (note: for these two samples the preparation steps were similar to those performed by Zelenay's group).

From the relative absorption area of each iron site and the absolute iron content in the final catalyst, one can estimate the relative abundance of each type of iron site. These values are given in Supplementary Table 4, and will be used in the discussion part to derive a mass-based maximum site density (MSD_max_).

X-ray photoelectron spectroscopy (XPS) was used to probe the near-surface nitrogen content of the Me–N–C catalysts. It confirmed the presence of a high fraction of nitrogen as required for the chemical coordination of the metal sites. Supplementary Figure 4 displays XPS survey spectra and N1s high resolution scans for all three catalysts. Supplementary Table 6 summarizes the related elemental composition. All three Me–N–C catalysts show distinct contributions of the Me–N coordination in their N1s fine scans and varying contributions of pyridinic, pyrrolic, graphitic and oxidic nitrogen. For reasons of comparison, the metal-free N–C catalyst was probed as well. Except for the metal species its elemental composition is similar to our Me–N–C catalysts. It should be noted that the contribution assigned as pyrrolic nitrogen is more pronounced in this catalyst, while the Me–N_*x*_–peak is missing.

X-ray absorption near edge spectroscopy (XANES) measurements were performed to characterize the Mn oxidation state in the two manganese-containing catalysts. In Supplementary Fig. 6, the XANES spectra of the Mn L_3,2_ edge of both Mn-containing samples are compared. The catalysts reveal similar fine structures of two main contributions A_1_, A_2_ that appear at 639 eV, 640.5 eV and 649 eV, 651 eV, respectively, for the L_3_ and L_2_ edges. In addition, there are slight contributions B at 642.5 eV for L_3_ (653 eV: L_2_-edge). The fine structure resembles that of MnPc/Au measured under an incident beam angle of 30°, which is close to our experimental conditions. These energies describe the charge transfer from Mn 2p_3/2_ (L_3_) and 2p_1/2_ (L_2_) states into hybridized Mn d-states^35^. It should be pointed out that the fine structure is pretty much different compared with manganese oxides^36^ leading to the conclusion that the manganese ions are indeed embedded in the N_*x*_ environment.

**Figure 4:**
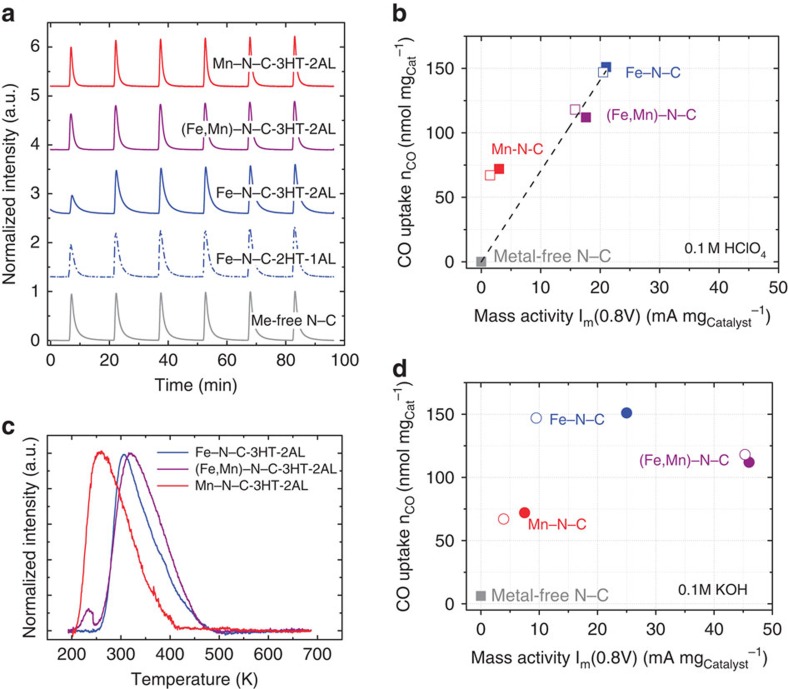
Results obtained from CO sorption. (**a**) Carbon monoxide (CO) pulse chemisorption profiles of N–C (blank catalyst; grey trace), Fe–N–C–2HT–1AL (dashed blue trace), Fe–N–C–3HT–2AL (blue trace), (Fe,Mn)–N–C–3HT–2AL (violet trace), Mn–N–C–3HT–2AL (red trace); CO uptake (nmol mg_catalyst_^−1^) versus ORR catalyst mass activity I_m_ at 0.8 V (mA mg_catalyst_^−1^) in (**b**) 0.1 M HClO_4_ and in (**d**) 0.1 M KOH (filled symbols: after 3HT–2AL, open symbols: after 2HT–1AL), (**c**) Normalized temperature-programmed desorption (TPD) profiles of Fe–N–C–3HT–2AL (blue line), (Fe,Mn)–N–C–3HT–2AL (violet line), and Mn–NC3HT–2AL (red line), Conditions: CO adsorption at 193 K, desorption ramp: 20 K min^−1^ to 693 K. a.u., arbitrary unit.

To quantify the density of active adsorption sites at the surface of the catalysts, CO pulse chemisorption experiments at 193 K followed by TPD of CO were carried out (see Supplementary Fig. 7 for instrumental details). Figure 4a reports the pulse chemisorption traces of all three final Me–N–C–3HT–2AL catalysts, as well as two reference catalysts. The reference samples comprise the metal-free N–C and the Fe–N–C–2HT–1AL catalyst, which is the mother sample of Fe–N–C–3HT–2AL.

The CO pulse chemisorption data provide two new important insights: first, there is no detectable adsorption of CO on the metal-free N–C sample, implying that CO adsorption on metal-free nitrogen functionalities is negligible or below our detection limits for the given experimental conditions. Second, the amount of adsorbed CO increased with the ORR activity. We are inclined to conclude that CO molecules adsorbed predominantly on surface sites relevant to ORR catalysis. This conclusion is further supported by the monotonic correlation between the molar amounts of adsorbed CO per mg_catalyst_ with the mass activities of the iron-based Me–N–C catalysts in acid in Fig. 4b. Previously unaddressed, this correlation and its monotonic character tend strong evidence to our claim of probing catalytically active sites in each sample.

To learn more about the chemical bonding of CO to catalytic surface sites, we conducted TPD experiments of the three Me–N–C catalysts (Fig. 4c). The TPD data show that the CO desorption temperature (and rate) is sensitive to the specific nature of the metal, starting at 210 and 260 K for Mn–N–C–3HT–2AL and Fe–N–C–3HT–2AL, respectively. The substantially higher desorption temperature of the iron-based catalyst suggests a larger desorption energy barrier, possibly related to a stronger CO binding on Fe-containing moieties what could be an indication of a more favourable interaction with O_2_ as well. Interestingly, our bimetallic catalyst (Fe,Mn)–N–C–3HT–2AL shows two separate desorption peaks. The slight down- and up-shift observed in the individual desorption peak temperatures of Mn and Fe sites in this bimetallic catalyst, respectively, seem to indicate some differences in their detailed CO desorption kinetics compared with the monometallic catalysts. Furthermore, comparing the experimental TPD peak integrals with the actual metal ratio of Fe:Mn=3:1 (Supplementary Table 3) suggests that most of the CO molecules appear to be adsorbed on Fe sites in the bimetallic (Fe,Mn)–N–C–3HT–2AL catalyst. Finally, our present TPD results reasonably explain why previous attempts to probe or block the catalytically active sites of non-precious Me-N–C ORR catalysts by CO failed^27^: those experiments were performed at room temperature throughout, which is why the interaction of CO with the metal active sites was too weak to enable chemical adsorption. Significantly lower temperatures are required. One might suspect that a further decrease in temperature might enable to probe even metal-free N–C sites that are ORR active in alkaline and at higher over potentials in acid.

## Discussion

We have applied two distinctly different analysis techniques that is CO adsorption and Mössbauer spectroscopy. Now, we address the quantification of mass-based surface active-site density, MSD and TOF_CO_, values based on accessible surface sites probed by CO chemisorption. The TOF_CO_ values derived from CO pulse chemisorption were calculated by dividing catalyst mass activities *I*_m_ at +0.8 V versus RHE by the molar amount of adsorbed CO molecules *n*_CO,Catalyst_ and the Faradaic constant F according to


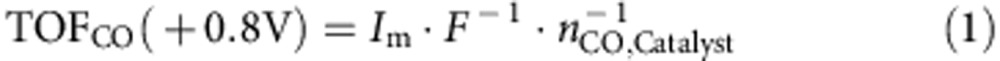


**Figure 5:**
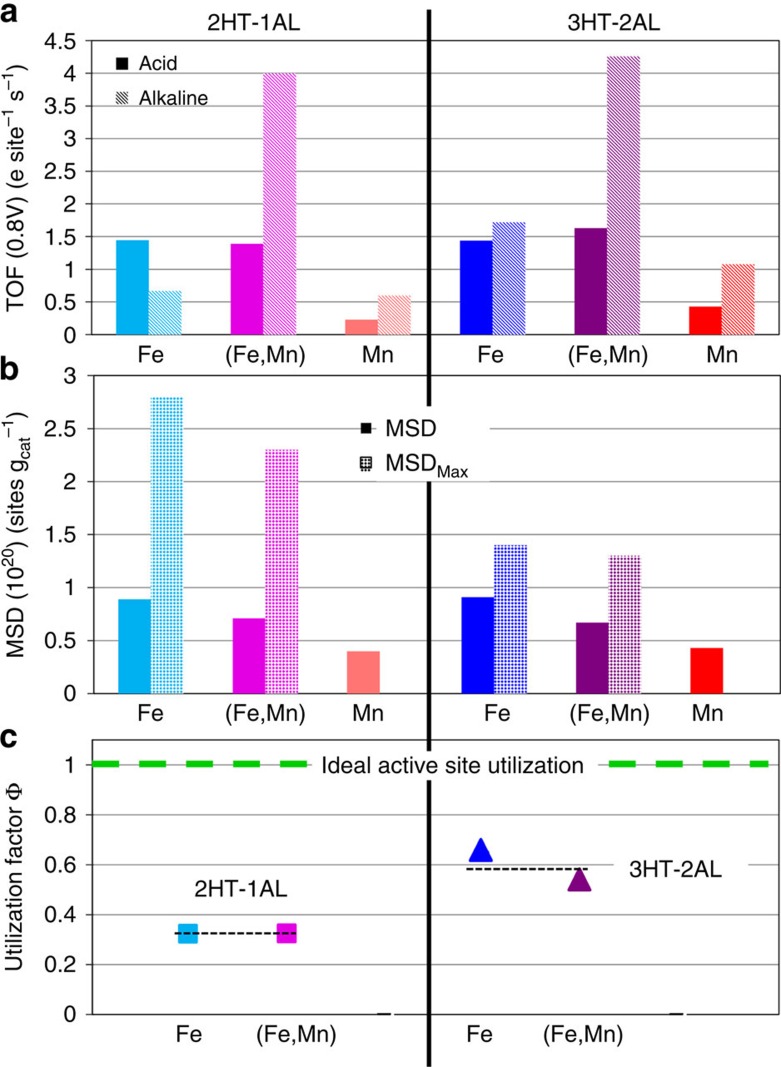
Comparison of catalytic parameters. (**a**) Comparison of the turn-over frequency (TOF) values as derived from CO chemisorption (TOF) in acid and alkaline electrolytes. (**b**) Comparison of the mass-based surface active and maximum site densities derived from CO chemisorption (MSD) and Mössbauer spectroscopy (MSD_Max_), respectively. (**c**) The active-site utilization factor Φ_CO/MBS_ as defined in equation 4 is plotted for the Fe-N-C and (Fe,Mn)–N–C catalysts with 3HT–2AL and with 2HT–1AL, its distance to the horizontal dashed line represents a quantitative measure of the promise of a particular catalyst material and catalyst preparation method (ideal utilization Φ_CO/MBS_=1).

The TOF_CO_ values in acid and alkaline are plotted as solid and hatched bars, respectively, in Fig. 5a. As shown in Fig. 4a, the metal-free N–C catalyst does not indicate any CO adsorption. Consequently, this method of calculating the TOF values is only applicable to Me–N–C catalysts. In addition, it should be noted that possibly our CO adsorption experiment was not performed at the saturation point, that is, it is possible that not all sites are probed by CO adsorption at −80 °C and lower temperatures would be required to reach saturation. Hence, the obtained TOFs display upper limits for these catalysts.

In acidic environment, the TOF_CO_ values were similar for both iron-containing catalysts (1.5 electrons per site per s). Hence, it appears that the bimetallic nature has no impact on the ORR characteristics in acid, while we have seen that it substantially improves the catalyst stability during ASTs in acidic electrolyte (Fig. 2). This observation is in line with previously reported trends by Wu *et al*.^5^,^9^,^37^, where bimetallic PANI–Me–C-derived catalysts (Me=Fe and Co) were more stable compared with the Fe-only ones^5^. Considering that both the Fe and the Mn monometallic catalysts showed similar final metal contents, while the ORR activity was significantly smaller for the Mn–N–C–3HT–2AL catalyst, we conclude that a smaller fraction of active sites in the Mn–N–C catalyst is surface accessible for O_2_ or CO molecules. In addition, its significantly smaller TOF is in line with theoretical calculations performed for MeN_4_ sites in pyridinic and pyrrolic environment^38^.

In alkaline environment, the TOF_CO_ values of metal sites exceeded nearly always those obtained in acidic environment, in accordance with earlier reports on pH effects in non-precious ORR catalysis^39^,^40^,^41^. The discrepancy between our optimized iron-containing catalysts (3HT–2AL) and Mn–3HT–2AL, however, is more distinct in alkaline environment (variation between 1.1 and 4.5 electrons per site per s). One reason might be given by the additional contribution of other sites that reveal a pronounced ORR activity in alkaline but not in acidic environment, as for instance nitrogen functionalities or quinone groups that might not be probed by CO under our given experimental conditions^42^,^43^,^44^,^45^,^46^. A comparison of our TOF values with literature is limited, as so far there were no TOF values based on a surface-sensitive probe. Reported values were derived from Mössbauer spectroscopy or just from the relation of ORR current densities towards the overall concentration of metal in these Me–N–C–catalysts^16^,^17^,^20^,^30^,^47^.

Comparing our values with those previously reported, we achieve one of the highest TOF values for NPMC. The only TOF value that exceeds ours is related to a binary active site composed of a high-spin NFeN_2+2_ site interacting with a protonated nitrogen group reported by Dodelet's group: NFeN_2+2_…NH^+^/C (TOF(0.8 V)≈10 electrons per site per s)^20^. This particular site, however, undergoes a very fast degradation due to anion binding that limits its applicability to the fuel cell technology so far^2^,^11^,^20^,^48^,^49^. Hence, we emphasis that our catalysts combine auspicious TOF values and a relatively stable performance. In addition, it should be noted that the real intrinsic TOF might even be higher as the accessibility of the active sites in our CO adsorption experiment might be significantly better compared with real operating conditions that limit O_2_ diffusion.

Beside the TOF, the density of active sites is another important parameter for the optimization of catalysts. If each catalytic active site is blocked by one CO molecule, the molar amount of adsorbed CO per mg_catalyst_ *n*_CO_ (in nmol mg^−1^) equals the molar concentration of active surface sites contributing to the ORR process. By multiplying the measured values with Avogardo's constant N_A_, we get the mass-based surface site density MSD (2).


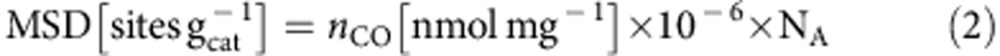


We would now like to compare our CO chemisorption derived mass-based site densities, MSD, with analogous mass-based site densities, derived from Mössbauer spectroscopy. However, we would like to point out the difference in the distinct probing characteristics of both methods: Mössbauer spectroscopy is a bulk method probing the total density of specific Fe sites, typically deviating from the number of Fe sites actually accessible for adsorption of O_2_ or CO molecules. Indeed, it was shown previously for Fe–N–C catalysts that fractions of D1-related sites are ‘hidden' in the bulk of the material and consequently do not contribute to the ORR activity^16^,^30^. As such, the amount of the D1-related FeN_4_ sites provides a measure of the maximum number of possible ORR active sites referred to as the maximum mass-based site density MSD_max_, given the perfect accessibility to all active FeN_4_ sites. Based on previous reported correlations between ORR activity and our D1 motif, we exclusively ascribe the ORR activity to the D1-related FeN_4_ species^16^,^17^,^30^. Furthermore, based on TPD results, we deduce that for (Fe,Mn)–N–C–3HT–2AL most of the ORR activity is related to the D1 motif. Accordingly, under the precondition of similar Debye–Waller factors, the maximum mass-based site density MSD_max_ can be calculated from Mössbauer spectroscopy by equation (3):


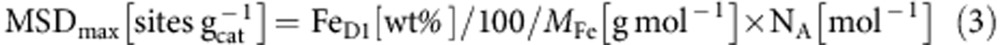


Here, Fe_D1_ [wt%] denotes the iron concentration of the D1 motif (Supplementary Table 4), M_Fe_ the molar mass of iron and N_A_ Avogardo's constant. Figure 5b compares these mass-based site densities of our CO chemisorption experiments with those that are derived from Mössbauer spectroscopy.

We would like to point out that the exclusive assignment of ORR activity to D1 was based on the following requirements that we demand for an ORR active site: (1) a significant contribution of the site to the ORR based on structure activity correlation and (2) the correlation should be of general nature and hold for differently prepared catalysts rather than just a single preparation route. A contribution of D2 can be excluded due to previous results^11^,^16^,^17^,^30^. The D3 and D4 motifs were not present in Me–N–C catalysts so far. A significant contribution of D4 to the ORR activity of these catalysts is excluded by the fact that this site is only present in the iron-based catalyst with a substantial amount. Hence, if it contributes to the ORR, the mass-related activity of Fe–N–C–3HT–2AL should be reasonably higher compared with (Fe,Mn)–N–C–3HT–2AL in both electrolytes. Notably, this is not the case. Taking into account, also the D3 motif would lead to even higher maximum mass-related site densities MSD_max_ in our catalysts (but consequently lower utilization factors, see below). For reasons of comparison, we have included two columns in Supplementary Table 7 that give the maximum mass-based site densities (MSD_max_) either considering all FeN_4_ sites or only the D1-related FeN_4_ sites.

To characterize the catalyst performance and their possible enhancement through optimizing the catalyst surface area and mass-based site density, we introduce the ratio


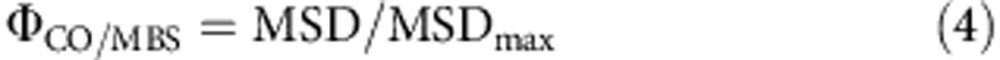


to represent an active-site utilization factor quantifying what ratio of the total number of catalytically active sites in a catalyst is actually accessible for the ORR. This utilization factor Φ_CO/MBS_ is plotted for the Fe-containing catalysts in Fig. 5c. Evidently, Φ_CO/MBS_ are very similar for each two catalysts after 2HT–1AL and 3HT–2AL, which supports the notion of Φ_CO/MBS_ representing an experimental meaningful descriptor for physically possible additional activity enhancements. To illustrate this, we point to the green dashed line in Fig. 5c with Φ_CO/MBS_=1, which represents the optimal utilization of Fe sites in Me–N–C catalysts. In this case, all active Fe sites in a catalyst would be accessible by O_2_ (and CO) and contribute to the ORR process. Similarly, Φ_CO/MBS_ offers projections of the maximum achievable catalyst current density of a specific family of catalysts, by dividing the experimentally observed mass activity by Φ_CO/MBS_. This is important as such projections allow a rapid and objective assessment and comparison of the theoretical promise of a specific preparation route or of specific precursors.

For the materials used here, our analysis uncovers that the mass activities could be further enhanced by a factor of 1.5x (for Fe–N–C–3HT–2AL) and 1.9x (for (Fe,Mn)–N–C–3HT–2AL) provided a suitable preparation. This enhancement could be realized by optimizing the catalyst dispersion such that all active sites are located on accessible pore surfaces. For a better evaluation of the utilization factor, Mössbauer spectroscopy was also applied to the two iron-containing catalysts with 2HT–1AL. Their TOFs, mass-based site densities and the utilization factors are given in Fig. 5, too. The Mössbauer spectra are shown in Supplementary Fig. 5. Both catalysts contain the same iron species in approximately similar ratios as after 3HT–2AL. The similar Mössbauer signatures indicate that there are no significant electronic changes induced by the HT and AL cycle. Consequently, the TOF values and mass-based surface active-site densities after 2HT–1AL and after 3HT–2AL are similar for both catalysts (Fe and (Fe,Mn)). In contrast, the maximum mass-based site densities derived from Mössbauer spectroscopy are significantly larger for 2HT–1AL compared with 3HT–2AL. This indicates that for these catalysts basically the ratio of surface accessible and hidden in the bulk active sites has changed, which is induced by the repeated HT and AL cycle. Furthermore, we would like to point out that due to the similarity in the preparation routine also the utilization factors are basically determined by the number and kind of HT and AL cycles. Our results indicate that the last HT–AL cycle was not beneficial for the iron-containing catalysts in terms of the maximum mass-based site density MSD_max_. Due to the AL that was involved not only inactive but to the same degree active sites were removed. Hence, the utilization of active sites should have been improved by another approach to enhance the ORR activity. In contrast, assuming by similarity of these three metal combinations similar Φ_CO/MBS_ factors for the Mn–N–C catalysts the change in the utilization factor going from 2HT–1AL to 3HT–2AL equals the increase in ORR activity (Φ enhancement: 1.9, ORR enhancement: 2.0).

Beside the importance of the utilization factor for the catalyst activity (and the number of sites contributing), we believe that the utilization factor will be an important descriptor for the catalyst stability in the general concept of heterogeneous catalysis. For example, the ‘stable' performance of the PANI–Fe–C catalyst in a potentiostatic test at 0.6 V, as described by Ferrandon *et al*., was related to a balance of active-site destruction and utilizing active sites that were previously hidden in the bulk^50^. Consequently, the utilization factor Φ was significantly smaller than 1 at the beginning of the experiment and should come close to one at the time where the activity starts to decrease. Or in other words, during FC application, the activity decreases as soon as active-site destruction, and the utilization of hidden sites is not balanced anymore.

In conclusion, our combined analysis of non-precious ORR catalysts using CO adsorption and Mössbauer spectroscopy offers a previously unachieved quantification of active-site density and intrinsic TOF activity descriptors. The two experimentally accessible site density values yielded an active-site utilization factor Φ_CO/MBS_ that serves as a quantitative descriptor for the promise and potential of a specific chosen preparation technique with respect to its activity and stability. As such, Φ_CO/MBS_ enables an accelerated screening of the potential of different catalyst preparation techniques and, hence, is of general importance for future advances in the development of Fe-containing non-precious ORR catalysts.

## Methods

### Synthesis

Synthesis of Me–N–C catalysts involved a multistep preparation that is schemed in Supplementary Fig. 1. For the Fe–N–C–3HT–2AL catalyst, 3 g FeCl_3_ was added into 2 ml of pure aniline (Sigma-Aldrich) dissolved in 250 ml 0.5 M HCl. This solution was kept stirring for 1 h at 4 °C. Thereafter, 5 g APS ((NH_4_)_2_S_2_O_8_), as the oxidant, was added drop wise to inform the *in situ* polymerization of aniline. Finally, Ketjenblack EC-300 J (0.4 g), which was treated first in 0.5 M HCl (at room temperature, 24 h), then refluxed in concentrated nitric acid (90 °C, 5 h) and subsequently washed with H_2_O was mixed with FeCl_3_/PANI under constant stirring. The mixture was kept under continuous stirring at room temperature for 48 h. The desired product was then refluxed and vacuum dried to obtain the precursor powder. To achieve the final catalyst, 3HT steps and 2ALs were performed (compare Supplementary Fig. 1). The first HT (pyrolysis) for 1 h and the second and third HTs for 3 h performed in N_2_ at 900 °C. Each AL was made at 90 °C in 2 M H_2_SO_4_ for 5 h, followed by filtration and rinsing with water (until neutralisation), whereas each preparation was finalized by a HT step. Hence the Fe–N–C–3HT–2AL had the following sequence of preparation steps: HT1−AL1−HT2−AL2−HT3.

MnCl_2_ was used as the metal precursor for Mn-based catalysts. For bimetallic catalyst, a molar ratio of Mn:Fe=1:1 was chosen as the optimal metal ratio. The nomenclature is similar to the iron-based catalysts.

The metal-free reference catalyst was prepared similarly, but without any metal chloride during the oxidative polymerization. In this case, the starting precursors went through a HT in N_2_ at 900 °C for 1+3 h (hence, the duration at 900 °C was similar compared with Fe–N–C–2HT–1AL).

### RDE experiments

The catalyst ink was prepared by mixing 15.7 mg of catalyst powder with 800 μl H_2_O, 150 μl isopropanol and 50 μl Nafion (5 wt%). This mixture was ultrasonicated with an US-finger for 15 min while the ink was placed in an ice bath. From this homogeneous ink, 10 μl were dropped on a glassy carbon disc with a diameter of 5 mm yielding a catalyst loading of 0.8 mg cm^−^^2^ for the NPMC. The Nafion to catalyst ratio was 1:4. Cyclic voltammetry was first performed at 10 mV s^−1^ in N_2_-saturated electrolyte at 1,500 r.p.m. Then, the solution was saturated with oxygen and linear sweep voltammetry was recorded with 10 mV s^−1^ at 1,500 r.p.m. Catalyst mass activity (*I*_m_) in units of mA mg_catalyst_^−1^ was calculated according to *I*_m_=*I*_kin_/*L*_catalyst_, where *L*_catalyst_ is the geometric catalyst loading (mg_catalyst_ cm^−2^_geo_) and *I*_kin_ is the geometric kinetic current density, which was estimated according to: *I*_kin_=*I*_0.8_ × *I*_lim_/(*I*_lim_−*I*_0.8_), using the geometric current density at 0.8 V versus RHE, *I*_0.8_, and the diffusion limited current density, *I*_lim_. Stability tests were performed up to 9,000 potential cycles in the potential range between 0.5 and 1.3 V versus RHE in N_2_-saturated electrolyte with the scan rate of 50 mV s^−1^ on stationary electrode. All activity and stability tests were performed at room temperature in 0.1 M HClO_4_ (pH 1) and 0.1 M KOH (pH 13). All the results have been compared with a reference Pt/C 20 wt% (E-TEK) catalyst with a geometric Pt loading of 10 μg_Pt_.

### PEM-FC measurements

The Fe–N–C–3HT–2AL and the (Fe,Mn)–N–C–3HT–2AL catalysts were tested in the fuel cell cathode to evaluate their activity and durability under selected polymer electrolyte fuel cell conditions. The catalyst ink was prepared by ultrasonically mixing the catalyst powder with Nafion suspension (DE 2029, 20 wt%) for 30 min. The as-prepared ink was applied to the gas diffusion layer by successive spray-painting until the cathode catalyst loading reached ca. 2 mg cm^−2^ for NPMC and 0.2 mg_Pt_ cm^−2^ as homogeneous catalyst films were formed. The Nafion content in the dry catalyst layer was maintained at ca. 50 wt%. At the anode, Pt 46 wt% (TKK Japan, 0.25 mgPt cm^−2^ loading) was used. The same spray-painting method was used to form the anode layer. The cathode and anode were hot pressed onto two sides of one piece of a Nafion NRE 212 membrane. The geometric membrane electrode assembly (MEA) area was 10.0 cm^2^. Fuel cell testing was carried out in a single cell. Pure hydrogen and oxygen, humidified at 80 °C were supplied to the anode and cathode (relative humidity 100%). Noteworthy, the same absolute pressure of 1 bars was applied to both electrodes. Fuel cell polarization plots were recorded using a fuel cell test station (Fuel Cell Technologies, Inc.). Recorded voltage was corrected for the resistance of the Nafion membrane as well as the recorded current was corrected for hydrogen cross over. The MEA voltage cycling tests were carried out by flow of nitrogen to the cathode in the voltage range of 0.6–1.0 V.

### CO pulse chemisorption and TPD

Pulse chemisorption and TPD experiments were performed utilizing a Thermo scientific TPD/R/O 1110 instrument (depicted in Supplementary Fig. 7) under a helium flow of 20 ml min^−1^ as carrier gas. For each catalyst, an amount of 100–150 mg was mounted between quartz wool in a quartz reactor assembling in a furnace (Supplementary Fig. 7). The temperature was measured at the sample position with a K-type thermocouple sealed in a quartz capillary. All samples were thermally pre-treated to remove any possible surface contamination, which can originate from adsorption of undesired molecules such as carbon species or water present in air. All adsorption processes in pulse chemisorption and TPD were carried out at 193±5 K by keeping the reactor in a bath of acetone and dry ice. In pulse chemisorption, CO consumption and in TPD the desorbed gas can be monitored through a thermal conductivity detector. The CO molecules are dragged by the carrier gas to thermal conductivity detector that measures the signal difference of the desorbed gas versus a reference flow. TPD experiments were carried out up to 693 K with a linear temperature ramp of 20 K min^−1^.

### ^57^Fe Mössbauer spectroscopy

Measurements were made for Fe–N–C and (Fe,Mn)–N–C catalysts after 3HT–2AL and 2HT–1AL. About 75–100 mg of each sample were filled into an aluminium sample holder with a diameter of 1.5 cm and a height of 3 mm. The sample holder is closed on both sides by Tesa tape. Hence, in the path of the γ-ray 10–30 μg_Fe-57_ cm^−2^ were present. Each sample was measured for about 10 days in two separate periods. The obtained spectra were qualitatively the same. To reduce noise the spectra were overlayed. Measurements were performed at room temperature with a 1,024 multichannel analyser equipped with a constant electronic drive system with triangular reference waveform (Halder Electronics, Germany). The Mössbauer relevant γ-radiation was provided by a 50 mCi Co/Rh source. Calibration of the velocity scale was made with respect to an α-Fe foil. Spectra were fitted with the programme Recoil assuming lorentzian line shape of all absorption sites.

### Determination of chemical composition

X-ray-induced photoelectron spectroscopy was carried out on an AXIS ULTRA DLD (Shimadzu) with monochromated Al K_α_ X-rays as the excitation source. Peak positions were internally referenced to the C1s peak at 284.6 eV. Fitting of the N1s fine scans were made with the UNIFIT software with 30% Lorentzian line form in the fitting process. In addition, the surface-near elemental composition was determined.

### Determination of metal contents

Initial and final metal contents were determined by optical emission spectroscopy using inductive-coupled plasma as ionization source, which was performed by Varian 715-ES. The samples were pre-treated by microwave at 180 °C to digest all moieties of the catalyst including carbon support. In a typical inductive-coupled plasma as ionization source sample preparation, ∼5 mg of the dry sample powder was weighted in a quartz tube and dissolved in a mixture of concentrated acids including: 2 ml sulphuric (98 wt%), 2 ml nitric (69 wt%) and 6 ml chloric (37 wt%) acids. Sample digesting was performed by Microwave (CEM SP-D Discover) at 120 °C for 10 min.

## Additional information

**How to cite this article:** Sahraie, N. R. *et al*. Quantifying the density and utilization of active sites in non-precious metal oxygen electroreduction catalysts. *Nat. Commun.* 6:8618 doi: 10.1038/ncomms9618 (2015).

## Supplementary Material

Supplementary Information Supplementary Figures 1-7, Supplementary Tables 1-7 and Supplementary References
