# Supplementary material for: Quantifying the density and utilization of active sites in non-precious metal oxygen electroreduction catalysts
Source: Nat Commun. 2015 Oct 21;6:8618. doi: 10.1038/ncomms9618 (PMC4639811; doi:10.1038/ncomms9618)
Supplement: Supplementary Information — Supplementary Figures 1-7, Supplementary Tables 1-7 and Supplementary References [file ncomms9618-s1.pdf]

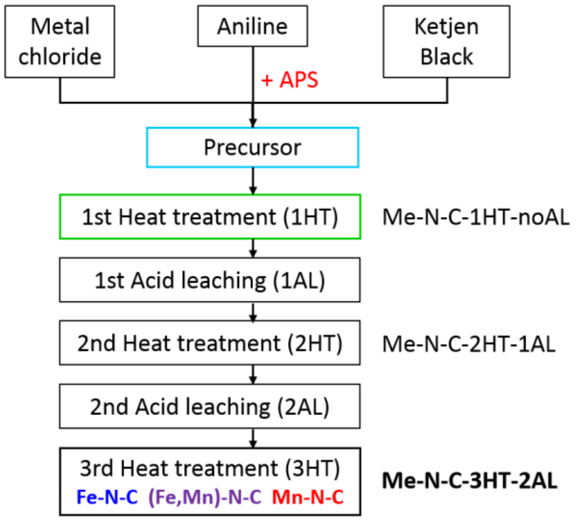

Supplementary Figure 1 | Catalyst preparation scheme. Scheme of the preparation route to obtain Me-N-C-nHT-(n-1)AL catalysts.

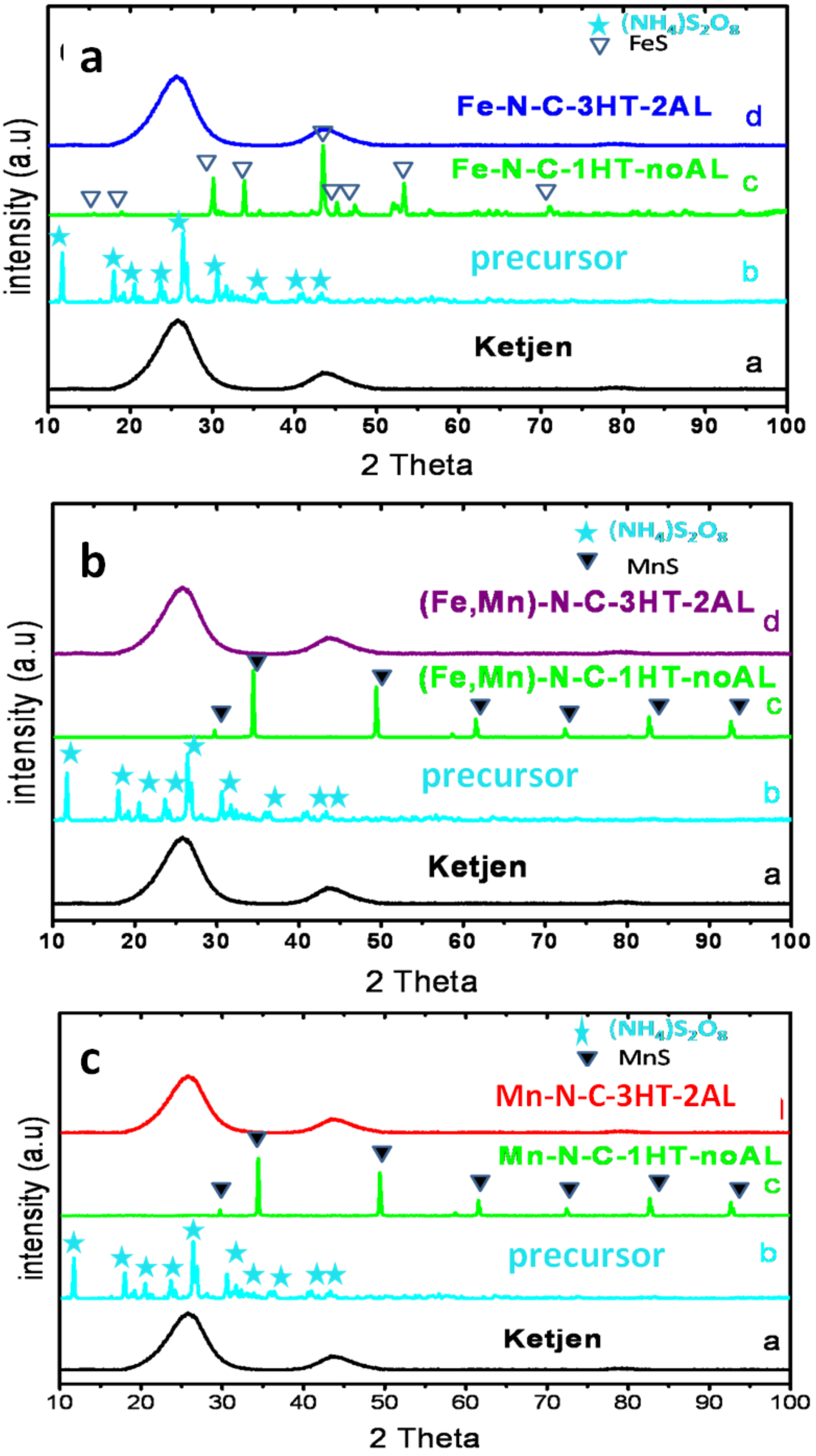

Supplementary Figure 2 | X-ray diffractograms of Me-N-C catalysts at their different preparation stages. XRD patterns of the final catalysts (-3HT-2AL) and after the first pyrolysis without acid leaching (-1HT-noAL) for a) Fe-N-C b) (Fe,Mn)-N-C and c) Mn-N-C. For reasons of comparison the XRD patterns of Ketjen Black and the corresponding Me-PANI-Ketjen precursors are also given.

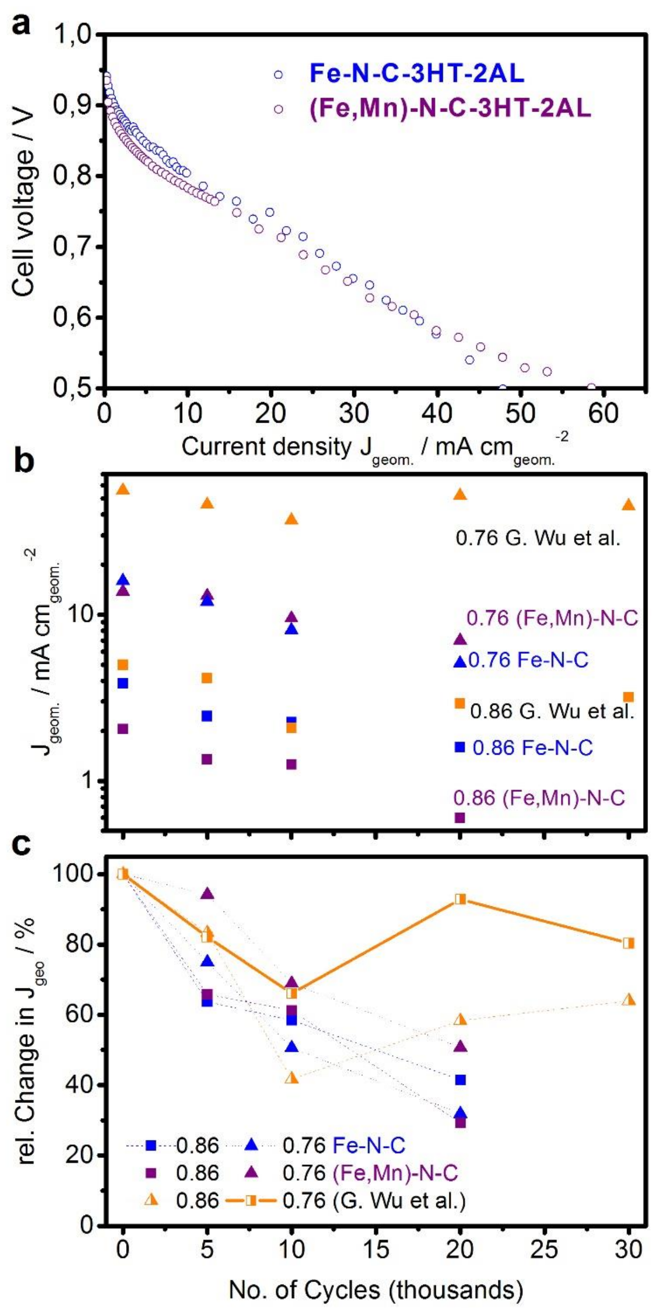

Supplementary Figure 3 | Fuel cell activity and stability testing. Fuel cell polarization curve and performance durability testing of Fe-N-C-3HT-2AL and (Fe,Mn)-N-C-3HT-2AL catalysts. (a) H_2-O_2 fuel cell polarization curves recorded at a loading of 2 mg cm^{-2} of non-PGM catalysts. Both tests used a Pt/C catalyst with a loading of 0.25 mg_{Pt} cm^{-2} at the anode; anode and cathode gas pressure: 1bar. (b) Long-term stability test of both catalysts at various potentials after potential cycling in nitrogen between 0.6 and 1.0 V. (c) Comparison of the relative change in current density to data provided in G. Wu et al., reference [1]. In that work pressure was at 1.0 bar and measurements were made with a cathode loading of 2 mg cm^{-2}.

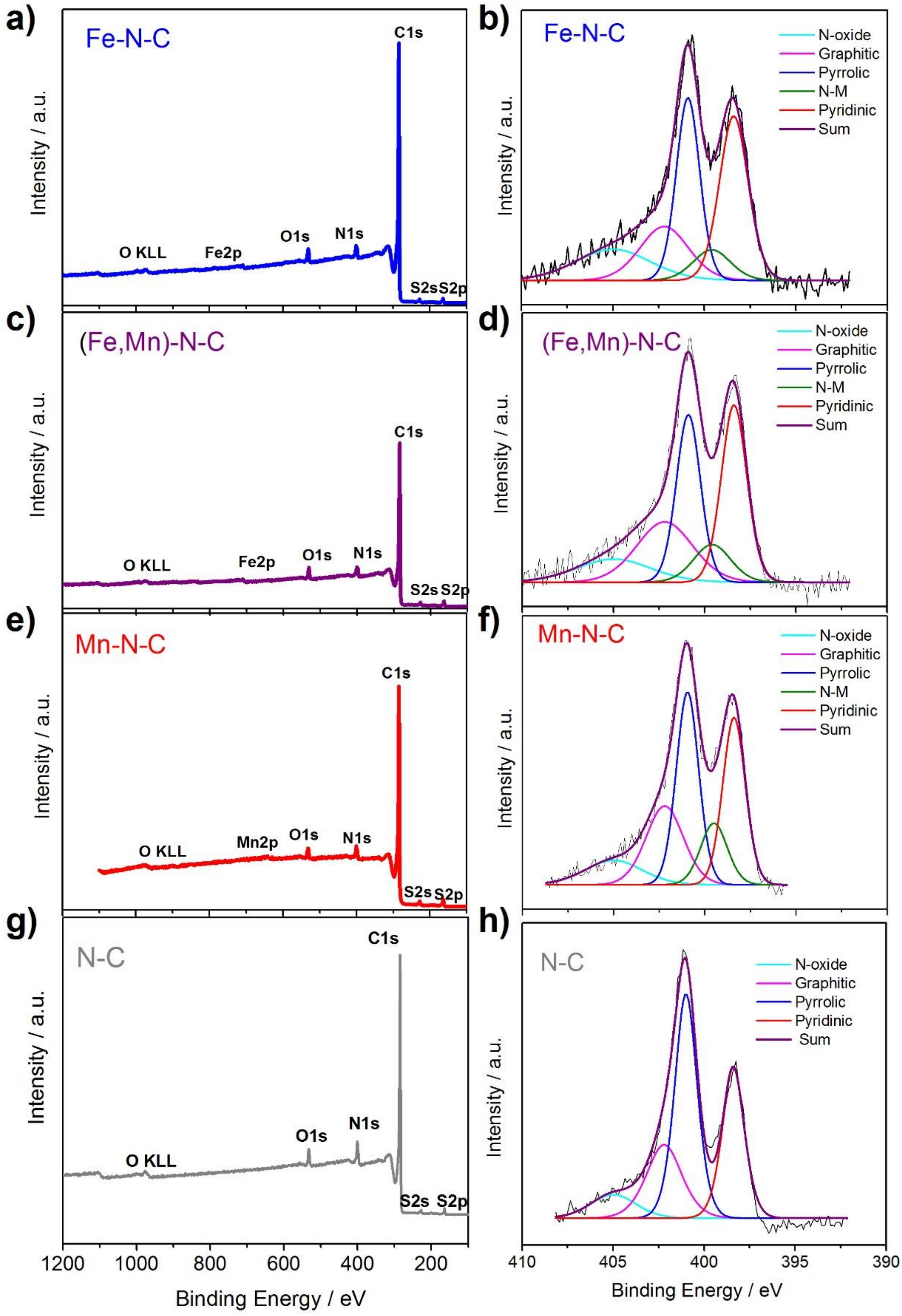

Supplementary Figure 4 | XPS survey scans and N1s fine scans. X-ray induced photoelectron spectroscopy (XPS) of the final conditioned catalysts (after 3HT-2AL) for a,b) Fe-N-C, c,d) (Fe,Mn)-N-C, e,f) Mn-N-C and g,h) metal-free N-C. On the left side (a ,c, e, g) and right side (b, d, f, h) survey scans and high resolution scans of the N1s- core level region including individual peak deconvolutions are provided, respectively.

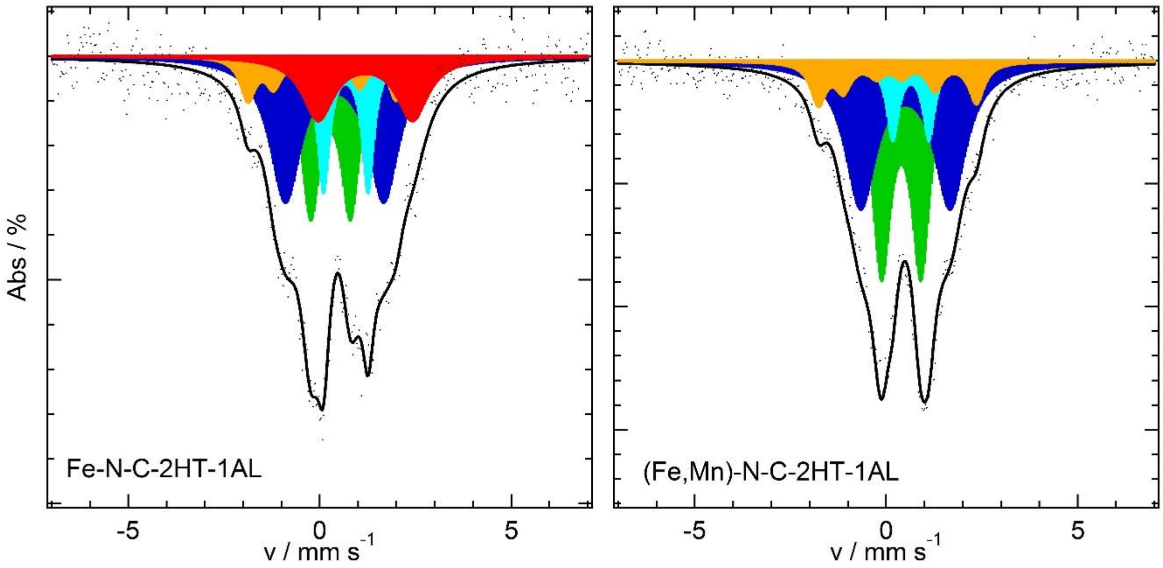

Supplementary Figure 5 | Mössbauer spectroscopy of iron-containing catalysts after 2HT-1AL. Reference measurements of the Mössbauer spectra of Fe-N-C-2HT-1AL (a) and (Fe,Mn)-N-C-2HT-1AL catalysts(b) for comparison to iron-containing catalysts after 3HT-2AL (Figure 3 of main manuscript).

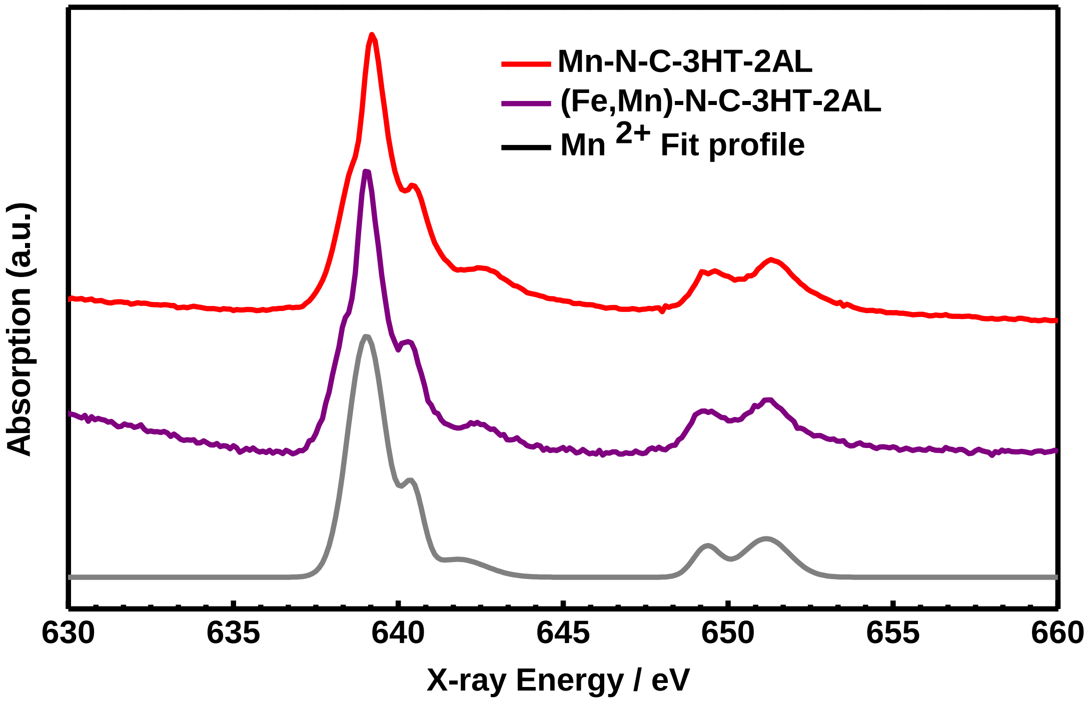

Supplementary Figure 6 | XANES profiles for Mn-containing catalysts after 3HT-2AL. XANES spectra of Mn L-edge for (Fe,Mn)-N-C-3HT-2AL and Mn-N-C-3HT-2AL.

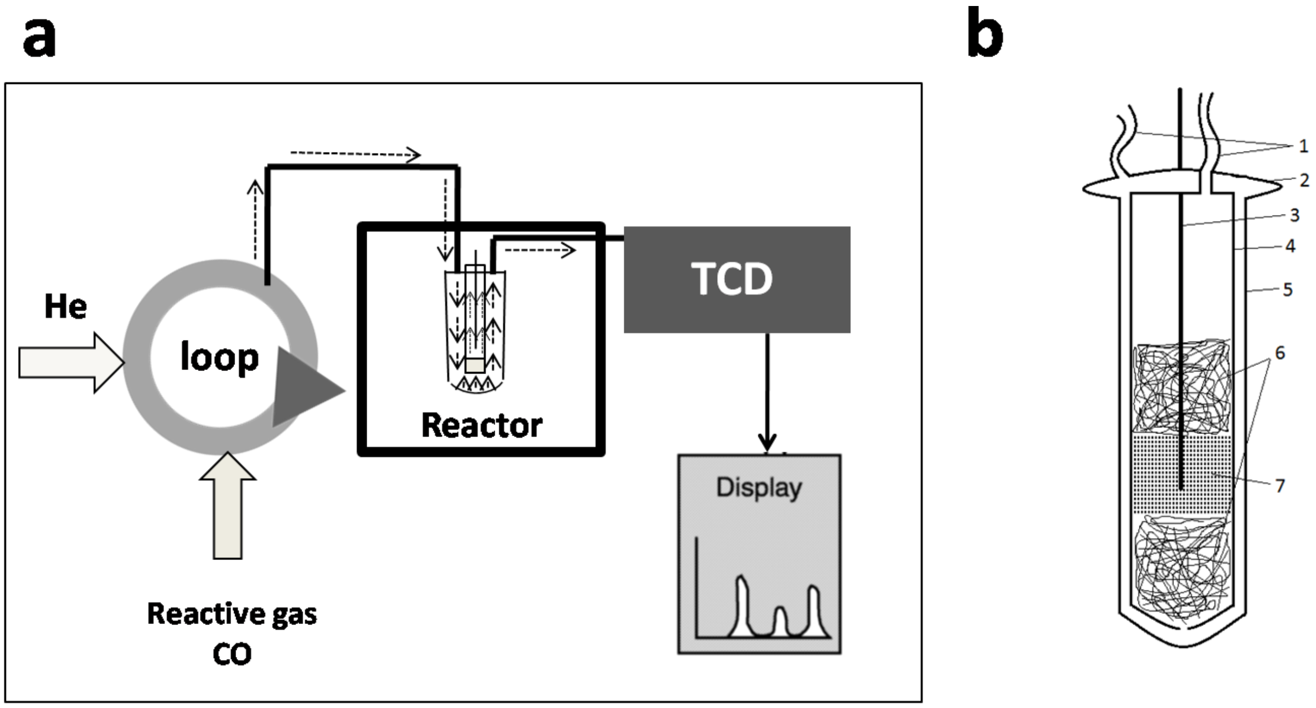

Supplementary Figure 7 | Schematic drawing of the experimental setup for pulsed chemisorption and desorption experiments. (a)Pulse chemisorption and temperature programmed desorption (TPD), carrier gas (He), thermoconductivity detector (TCD) instrument, (b) Schematic of reactor (right): 1) gas inlet/outlet, 2) sample holder cap, 3) thermocouple, 4) internal bulb, 5) external bulb, 6) glass wool, 7) sample (powder).

Supplementary Table 1 | RDE results for 0.1M $HClO_4$.

Summary of Rotating disk electrode (RDE) results in terms of onset potential at 0.1 mA cm^{-2}, half-wave potential, kinetic current density and mass activity at 0.8V for pH 1 $HClO_4$. All mono- and bimetallic catalysts were measured in O_2-saturated electrolyte (0.1 M $HClO_4$, pH 1) with 10 mV s^{-1} scan rate, at 1500 rpm, RT. 3HT-2AL= after 3rd heat treatment and 2nd acid leaching. 2HT-1AL= after 2nd heat treatment and 1st acid leaching. Experimental errors are indicated.

catalyst	Acid electrolyte / 0.1 M $HClO_4$			
	**E_{onset} (@0.1 mA cm^{-2})** V vs. RHE	**$E_{1/2}$** V vs. RHE	**kinetic current density (@0.8V)** mA cm^{-2}	**Mass activity (@0.8V)** mA $mg_{catalyst}^{-1}$
**Fe-N-C-2HT-1AL**	0.92 ± 0.02	0.81 ± 0.03	16.4 ± 0.7	20.5 ± 0.9
**Fe-N-C-3HT-2AL**	0.94 ± 0.01	0.84 ± 0.02	16.8 ± 0.8	21.0 ± 1.0
**(Fe,Mn)-N-C-2HT-1AL**	0.90 ± 0.02	0.80 ± 0.02	12.6 ± 0.9	15.8 ± 1.1
**(Fe,Mn)-N-C-3HT-2AL**	0.92 ± 0.01	0.82 ± 0.02	14.1 ± 0.6	17.6 ± 0.8
**Mn-N-C-2HT-1AL**	0.88 ± 0.01	0.69 ± 0.03	1.2 ± 1.0	1.5 ± 1.2
**Mn-N-C-3HT-2AL**	0.90 ± 0.02	0.77 ± 0.01	2.4 ± 0.7	3.0 ± 0.9
**Pt/C 20 wt%_ETEK**	0.95 ± 0.03	0.86 ± 0.01	21.3 ± 0.7	425 ± 0.9

Supplementary Table 2 | RDE results for 0.1 KOH.

Summary of Rotating disk electrode (RDE) results in terms of onset potential at 0.1 mA cm^{-2}, half-wave potential, kinetic current density and mass activity at 0.8V for pH 13 KOH. All mono- and bimetallic catalysts were measured in O_2-saturated electrolyte (0.1 M KOH, pH 13) with 10 mV s^{-1} scan rate, at 1500 rpm, RT. 3HT-2AL= after 3rd heat treatment and 2nd acid leaching. 2HT-1AL= after 2nd heat treatment and 1st acid leaching. Experimental errors are indicated.

catalyst	Alkaline electrolyte / 0.1 M KOH			
	**E_{onset} (@0.1 mA cm^{-2})** V vs. RHE	**$E_{1/2}$** V vs. RHE	**kinetic current density (@0.8V)** mA cm^{-2}	**Mass activity (@0.8V)** mA $mg_{catalyst}^{-1}$
**Fe-N-C-2HT-1AL**	0.92 ± 0.02	0.82 ± 0.02	7.6 ± 0.8	9.5 ± 1.0
**Fe-N-C-3HT-2AL**	0.94 ± 0.02	0.85 ± 0.01	20.0 ± 0.7	25.0 ± 0.9
**(Fe,Mn)-N-C-2HT-1AL**	0.97 ± 0.01	0.87 ± 0.02	36.2 ± 0.6	45.3 ± 0.7
**(Fe,Mn)-N-C-3HT-2AL**	0.98 ± 0.01	0.90 ± 0.01	36.8 ± 0.4	46.0 ± 0.5
**Mn-N-C-2HT-1AL**	0.90 ± 0.03	0.74 ± 0.02	3.1 ± 0.6	3.9 ± 0.7
**Mn-N-C-3HT-2AL**	0.92 ± 0.04	0.79 ± 0.02	6.0 ± 0.6	7.5 ± 0.8
**Pt/C 20 wt%_ETEK**	0.95 ± 0.01	0.84 ± 0.01	11.8 ± 0.8	236 ± 1.0

Supplementary Table 3 | Change of metal content with the different preparation steps.

Metal contents in precursor (nominal) and after 2HT-1AL and 3HT-2AL steps.

Catalyst	Nominal Metal content / wt%	Me after 2HT-1AL / wt%	Me after 3HT-2AL / wt%
**Fe-N-C**	27	12	6
**(Fe,Mn)-N-C**	Fe: 13.5/Mn: 13.5	Fe: 6 / Mn: 3	Fe: 3 / Mn: 1
**Mn-N-C**	27	9	5

Supplementary Table 4 | Summary of the Mössbauer fit results.

Summary of the average Mössbauer parameters for the iron sites in Fe-N-C-3HT-2AL and (Fe,Mn)-N-C-3HT-2AL. For both catalysts the relative absorption areas and the estimated contents of iron in the different modification for each catalyst after 3HT-2AL are given. This estimate was made under the assumption of similar Debye-Waller factors. Errors are given in parentheses, f indicates a fixed value.

Mössbauer signature					Fe-N-C-3HT-2AL		(Fe,Mn)-N-C-3HT-2AL		Assignment
Site	δ_{iso}	ΔE_Q	fwhm	H_0	A	Fe	A	Fe	
	/ mm s^{-1}			/ T	/ %	/ wt%	/ %	/ wt%	
D1	0.32 (0.03)	0.94 (0.03)	0.57 (0.03)	-	22.1 (1.4)	1.33 (0.22)	38.9 (5.6)	1.17 (0.29)	ORR-active FeN_4-site (Fe^{2+}, low spin) [2]
D2	0.49 (0.03)	2.23 (0.09)	1.0 (f)	-	44.4 (1.8)	2.66 (0.37)	37.1 (3.6)	1.11 (0.22)	similar to FePc (Fe^{2+}, intermediate spin) [2]
D3	0.63 (0.06)	0.97 (0.05)	0.35 (0.07)	-	7.3 (1.2)	0.44 (0.12)	14.3 (4.7)	0.43 (0.18)	$(NFe^{III}N_4)-O_2$, [3]
D4	1.24 (0.15)	2.58 (0.29)	1.0 (f)	-	9.3 (1.8)	0.56 (0.16)			XY-FeN_4 (X,Y: weak ligands like O and/or N) [4]
Sext	0.19 (0.06)	-	0.5 (f)	12.2 (0.4)	17.0 (1.6)	1.02 (0.20)	9.7 (2.7)	0.29 (0.11)	oxidized Fe particles [5]

Supplementary Table 5 | Comparison of the Mössbauer parameters of doublet D4 with literature reports.

	δ_{Iso} / mm s^{-1}	ΔE_Q / mm s^{-1}	Assignment
**D4 site in this paper**	**1.24**	**2.58**	**sixfold coordinated $Fe^{II}N_4$ site**
average values for $NFe^{II}N_{2+2}\ldots NH^+$	0.99	2.13	
D3 in Ref. 4	1.01	2.32	fivefold coordinated $NFe^{II}N_4\ldots NH^+$
D3 in Ref. 6	0.98	1.94	fivefold coordinated $NFe^{II}N_4\ldots NH^+$

Supplementary Table 6 | Surface-near elemental composition.

XPS-based elemental composition (at%= atomic percent) for mono- and bimetallic catalysts after 3HT-2AL.

	**Fe-N-C-3HT-2AL**	**(Fe,Mn)-N-C-3HT-2AL**	**Mn-N-C-3HT-2AL**
**C1s (at%)**	91.72	89.22	90.11
**N1s (at%)**	4.06	5.05	4.42
**Fe2p (at%)**	0.33	0.58	-
**Mn2p (at%)**	-	0.09	0.50
**S2p (at%)**	0.92	1.98	1.92
**O1s (at%)**	2.98	3.08	3.05

Supplementary Table 7 | Turn-over frequencies and mass-based site densities for the different catalysts.

Summary of TOF values and mass-based site densities derived from CO adsorption as well as maximum mass-based site densities determined from Mössbauer spectroscopy for the iron-containing catalysts.

	TOF(0.8V)$_{CO}$ / electrons site^{-1} s^{-1}		Mass-based site densities MSD (·10^{20}) / sites g^{-1}		
			MSD (from CO ads.)	MSD$_{Max}$ (from Mössbauer)	
	**0.1M HClO$_4$**	**0.1M KOH**		**all FeN$_4$**	**only FeN$_4$ (D1)**
Fe-N-C-2HT-1AL	1.4	0.7	0.89	10.8	2.8
**Fe-N-C-3HT-2AL**	1.4	1.7	0.91	5.4	1.4
(Fe,Mn)-N-C-2HT-1AL	1.4	4.0	0.71	5.7	2.3
**(Fe,Mn)-N-C-3HT-2AL**	1.6	4.3	0.67	2.9	1.3
Mn-N-C-2HT-1AL	0.2	0.6	0.40	-	-
**Mn-N-C-3HT-2AL**	0.4	1.1	0.43	-	-

Supplementary References

1. Wu, G., More, K. L., Johnston, C. M. & Zelenay, P. High-Performance Electrocatalysts for Oxygen Reduction Derived from Polyaniline, Iron, and Cobalt. *Science* **332**, 443-447 (2011).
2. Kramm, U. I. *et al.*Influence of the Electron-Density of FeN_4-Centers towards the Catalytic Activity of Pyrolysed FeTMPPCl-Based ORR-Electrocatalysts. *J. Electrochem. Soc.***158**, B69-B78 (2011).
3. Roelfes, G. *et al.* End-on and side-on peroxo derivatives of non-heme iron complexes with pentadentate ligands: Models for putative Intermediates in biological iron/dioxygen chemistry. *Inorganic Chemistry* **42**, 2639-2653 (2003).
4. Kramm, U. I., Herranz, J., Larouche, N., Arruda, T. M., Lefevre, M., Jaouen, F., Bogdanoff, P., Fiechter, S., Abs-Wurmbach, I., Mukerjeec, S. and Dodelet, J. P. Structure of the catalytic sites in Fe/N/C-catalysts for O2-reduction in PEM fuel cells. *Phys. Chem. Chem. Phys.*14, 11673–11688 (2012).
5. Greenwood, N. N. & Gibb, T. C. *Mössbauer Spectroscopy*. 1 edn, Vol. 1 (Chapman and Hall Ltd., 1971)
6. Kramm, U. I., Lefèvre, M., Larouche, N., Schmeisser, D. & Dodelet, J.-P. Correlations between Mass Activity and Physicochemical Properties of Fe/N/C Catalysts for the ORR in PEM Fuel Cell via 57Fe Mössbauer Spectroscopy and Other Techniques. *Journal of the American Chemical Society* **136**, 978-985 (2014).
